# The relationship between family physical activity environment and mental health in high school students: the chain mediating role of parent–child relationship and exercise behavior

**DOI:** 10.3389/fpubh.2025.1719426

**Published:** 2026-01-08

**Authors:** Xuezhen Feng, Enwei Xu

**Affiliations:** College of Educational Science, Xinjiang Normal University, Urumqi, China

**Keywords:** family physical activity environment, mental health, parent–child relationship, exercise behavior, high school students

## Abstract

**Introduction:**

Adolescence is a high-incidence period for mental health problems. Evidence suggests that the family physical activity (PA) environment may shape adolescents’ psychological well-being, but the underlying mechanisms remain unclear. This study examined whether the parent–child relationship and adolescents’ own exercise behaviour serially mediate the association between the family PA environment and mental health among Chinese seniorhigh-school students.

**Methods:**

A cross-sectional survey was conducted among 2,049 senior-high-school students (mean age = 15.7 ± 1.2 years; 52 % girls) recruited from six provinces in China. Validated instruments were used to assess the family PA environment (Family Physical Activity Environment Questionnaire), mental health problems (Mental Health Diagnostic Test), parent–child relationship quality (Parent–Child Relationship Scale), and exercise behaviour (Physical Activity Rating Scale). Structural equation modelling was employed to test direct and indirect effects, with sex, age, and socioeconomic status as covariates.

**Results:**

The family PA environment was significantly and positively associated with mental health (β = 0.31, *p* < 0.001). The parent–child relationship and exercise behaviour each independently mediated this association (indirect effect β = 0.09, 95 % CI 0.06–0.12; β = 0.07, 95 % CI 0.04–0.10, respectively). A significant chain-mediating effect was also observed (β = 0.05, 95 % CI 0.03–0.07), indicating that a supportive family PA environment improved relationship quality, which in turn promoted exercise behaviour and subsequently better mental health. The total mediated effect accounted for 41 % of the overall association.

**Discussion:**

Findings underscore the family PA environment as a critical determinant of adolescents’ mental health. Intervention programmes that simultaneously strengthen family-based physical activity and parent–child relationship quality may confer synergistic benefits for adolescent psychological well-being.

## 1 Introduction

Adolescents aged 15 to 18, generally referred to as high school students, are in a critical transitional period from adolescence to adulthood and represent the backbone of future society. As future builders of the nation and carriers of national expectations, their holistic development is of strategic importance for realizing the vision of national rejuvenation (1). International research indicates that the overall prevalence of psychological disorders among high school students is relatively high (2). For instance, a meta-analysis covering multiple countries reported that the detection rate of emotional and behavioral problems among adolescents is substantial, making this issue a global public health concern.

Family systems theory emphasizes that the family is the primary context for individual physical and psychological development, and the degree to which family functions are fulfilled is closely related to adolescents’ mental health. Dysfunction in core family functions—such as emotional bonding, communication, and support—can easily lead to internalizing problems (e.g., anxiety, depression) and externalizing problems (e.g., aggression) in adolescents (3). In the context of contemporary society, adolescents face emerging challenges including increased sedentary behavior and excessive electronic device use (4), which further exacerbate mental health risks and underscore the need for effective family-based interventions. With rapid social changes, traditional family structures and functions are increasingly challenged. In many families, educational practices prioritize academic achievement over holistic development, with insufficient attention and support for physical activity. The family physical activity environment, as an important context for promoting adolescent health, has attracted international attention. However, in practice, its value is often under-recognized and under-invested (5).

In international studies, family engagement in physical activity is regarded as a key factor in promoting adolescent mental health. For example, research has shown that joint participation in physical activities between parents and children not only enhances adolescents’ physical activity levels but also fosters family cohesion and positive interaction, thereby buffering psychological stress and promoting emotional regulation (6). Moreover, parental role modeling and supportive attitudes toward physical activity have been positively associated with adolescents’ psychological adjustment. However, current research exhibits significant limitations. While recent studies have explored individual pathways—such as intergenerational transmission of physical activity patterns (7) or the role of parent–child attachment in mental health (8)—they have failed to examine the sequential mechanisms through which family physical activity environment influences mental health via both relational and behavioral pathways. This represents a critical gap in understanding how family environmental factors translate into psychological outcomes through interconnected mediating processes.

The present study introduces substantial theoretical innovation by proposing and testing a chain mediation model (FPAE → Parent–Child Relationship → Exercise Behavior → Mental Health). This model provides two key advancements: first, it reveals how family physical activity environment strengthens parent–child bonds through shared activities, subsequently promoting sustained exercise behavior; second, it demonstrates how improved parent–child relationships and increased physical activity jointly contribute to mental health enhancement. This comprehensive framework moves beyond existing fragmentary approaches to offer an integrated understanding of the mechanisms underlying family physical activity environment’s mental health benefits. Against this backdrop, the present study adopts a family education perspective to examine the effects and mechanisms of the family physical activity environment on high school students’ mental health. Through empirical analysis, this study aims to provide a scientific basis for parents and educators, thereby advancing the development of supportive family physical activity environments and contributing to the improvement of adolescent mental health and holistic development.

## 2 Literature review and research hypotheses

### 2.1 Family physical activity environment and mental health of high school students

To clarify the concept of the family physical activity environment, it is necessary to distinguish between family sports and the family physical activity environment. While prior research conceptualizes the family physical activity environment (FPAE) as a multidimensional construct encompassing behavioral, attitudinal, and cultural dimensions, the present study focuses specifically on its empirically measurable core components. Based on previous research, the present study defines the family physical activity environment as a comprehensive system encompassing parental behavioral support, positive attitudinal guidance, and a shared cultural atmosphere toward physical activity within the family. A favorable family physical activity environment can effectively stimulate adolescents’ interest in sports and cultivate lifelong habits of physical activity (9). However, consistent with recent empirical consensus (5, 6), this study operationally defines FPAE through two proximal dimensions—parental role modeling and parental support—which are theoretically most relevant to initiating behavioral change in high school students. Considering that adolescents are developmentally immature and economically dependent on their families, the operational definition of the family physical activity environment in this study emphasizes parents’ intentional and conscious educational practices in relation to their children’s physical activity. These practices are conceptually categorized into role modeling (knowledge transmission, demonstration, and joint participation) and parental support (encompassing both psychological encouragement and material assistance), forming the basis for our empirical measurement.

The present study integrates Family Functioning Theory as its primary theoretical framework, which provides a coherent lens through which to examine how family physical activity environment influences adolescent mental health. Family functioning theory is one of the most influential frameworks for explaining how family dynamics affect adolescent mental health. This theory has two orientations: Outcome orientation, which describes family functioning in terms of specific attributes, such as emotional connections among members, family rules, communication patterns, and the ability to cope with external events (10). Process orientation, which evaluates family functioning in terms of its fulfillment of essential tasks, such as providing environmental conditions for members’ physiological, psychological, and social development (11).

Both orientations suggest that better family functioning is associated with higher levels of adolescent mental health. Within this theoretical framework, parental role modeling and support in physical activity represent concrete behavioral manifestations through which family functioning influences adolescent development. The family physical activity environment mobilizes and strengthens family functions by fostering trust, understanding, and cooperation among family members, thereby facilitating smoother fulfillment of family functions. Empirical evidence has confirmed the association between two dimensions of the family physical activity environment—parental role modeling and parental support—and adolescent mental health. When parents accompany their children, adolescents benefit psychologically not only from outdoor sports but also from more sedentary leisure activities (12, 13). It should be noted, however, that while the theoretical framework and hypotheses primarily emphasize the direction from family environment to adolescent outcomes, the relationships among these variables may be more complex. Recent research highlights the potential for bidirectional effects, where adolescents’ psychological characteristics and mental health status can also influence family dynamics and parental behaviors (14, 15). Based on this evidence, Hypothesis 1 (H1) is proposed: The family physical activity environment has a significant positive predictive effect on students’ mental health during the high school education stage.

### 2.2 The mediating role of parent–child relationship in the association between family physical activity environment and high school students’ mental health

The process-oriented perspective of family functioning theory suggests that it is not the structural characteristics of the family system per se, but rather the processes through which family functions are realized, that directly influence individuals’ emotional problems and overall well-being. Among these processes, communication is regarded as one of the core dimensions in evaluating family functioning (16). This theoretical foundation is complemented by attachment theory, which together provide a more nuanced understanding of parent–child relationship dynamics. The parent–child relationship refers to a bidirectional and interactive interpersonal bond between parents and children, established within the family context on the basis of biological, adoptive, or legal ties. It is not a unidimensional concept but rather a dynamic system encompassing emotional, behavioral, and cognitive components, characterized by emotional depth, stability, and enduring influence (14).

Empirical studies have demonstrated that open and positive parent–child relationships enhance adolescents’ life satisfaction while reducing the risk of psychological problems. According to cognitive internalization theory, the internalization of interpersonal interactions plays a crucial role in shaping individuals’ cognitive and behavioral development (17, 18). Within the family setting, parents who maintain an active lifestyle and engage in regular physical activity tend to adopt a more positive life attitude. Such parents are more likely to build close and non-authoritarian relationships with their children, fostering high-quality, bidirectional communication and allowing children to experience greater parental understanding and support, which in turn promotes their psychological well-being (19). Notwithstanding the primary focus on parental influence, contemporary research acknowledges that child-specific factors, including temperament and psychological dispositions, can significantly shape relationship quality. For instance, adolescents with higher levels of anxiety or neuroticism may evoke different parenting responses, thereby influencing the overall parent–child dynamic (14).

Based on this evidence, Hypothesis 2 (H2) is proposed: Parent–child relationship mediates the association between the family physical activity environment and high school students’ mental health.

### 2.3 The mediating role of exercise behavior in the association between family physical activity environment and high school students’ mental health

Exercise behavior refers to physical activities of a certain duration, frequency, and intensity that individuals engage in during their leisure time (20). A large body of research has shown that participation in physical activities can effectively reduce stress, depressive symptoms, social anxiety, and loneliness. A supportive family physical activity environment can significantly enhance adolescents’ intrinsic motivation for physical activity, while parental approaches to sports education have also been found to facilitate children’s participation in exercise (21).

Self-determination theory and the socio-ecological model are strategically integrated here to provide complementary explanatory frameworks for understanding the mediating role of exercise behavior. Self-determination theory and the socio-ecological model provide explanatory frameworks for understanding the mediating role of exercise behavior. Self-determination theory posits that the fulfillment of three basic psychological needs—autonomy, relatedness, and competence—enables the internalization of extrinsic motivation, fostering autonomous motivation (22). Within the family physical activity environment, parents can stimulate and cultivate adolescents’ intrinsic motivation for exercise through both emotional and behavioral support, thereby encouraging their engagement in physical activity and fulfilling basic psychological needs, which in turn contributes to better mental health (23).

The socio-ecological model highlights the multidimensional factors through which family environments influence adolescents’ exercise behavior and mental health. For instance, the physical home environment may exert both positive and negative influences: the widespread use of electronic devices may increase sedentary behaviors, heightening the risk of internet addiction and associated behavioral problems. Conversely, a supportive family physical activity environment may mitigate these adverse effects, thereby promoting exercise behavior and improving mental health (4). Based on this evidence, Hypothesis 3 (H3) is proposed: Exercise behavior mediates the association between the family physical activity environment and high school students’ mental health.

### 2.4 The chain mediating role of parent–child relationship and exercise behavior

Within the family physical activity environment, parent–child relationship and exercise behavior are also closely interrelated. Positive parent–child relationships strengthen the intergenerational transmission of physical activity behaviors. This section synthesizes intergenerational transmission theory with the previously established theoretical frameworks to articulate a coherent sequential mechanism. According to intergenerational transmission theory and vertical transmission models, three primary pathways—parental education and communication, children’s behavioral imitation, and genetic inheritance—contribute to the intergenerational transmission of behaviors and values (24). For example, research has shown that mothers’ engagement in home-based physical activity has a direct intergenerational transmission effect, and this effect is further reinforced by a positive parent–child relationship (7).

Recent studies have further clarified the sequential mechanism through which a favorable family physical activity environment exerts its influence on adolescents’ exercise behavior and mental health. A supportive and communicative parent–child relationship serves as a psychological bridge that facilitates adolescents’ internalization of parental behavioral models (14, 25). In line with Self-Determination Theory, high-quality parent–child interactions characterized by warmth, autonomy support, and open communication satisfy adolescents’ basic psychological needs for autonomy, competence, and relatedness, thereby fostering intrinsic motivation for physical activity. Empirical evidence suggests that adolescents who perceive higher levels of parental understanding and emotional responsiveness are more likely to engage in autonomous and sustained exercise behaviors, whereas direct parental encouragement without relational closeness often leads to external or short-term compliance (25).

Therefore, improvements in the parent–child relationship can be viewed as the necessary psychological foundation that precedes and enables the development of adolescents’ exercise behavior. In this sequential process, the family physical activity environment first enhances communication quality and emotional closeness within the family (FPAE → P–CR), which subsequently promotes adolescents’ willingness to participate in physical activity (P–CR → EB) and, through this behavioral pathway, further contributes to improved mental health (EB → MH). This theoretically grounded order reflects a process of emotional internalization and behavioral consolidation rather than a simultaneous or reciprocal effect, thereby providing a more coherent causal chain consistent with current evidence on family-based health behavior formation (6, 14). While the proposed sequential model emphasizes the direction from family environment through relationship quality to behavior, it is important to acknowledge the theoretical possibility of reciprocal influences. Future longitudinal research would benefit from examining potential feedback loops where exercise participation might subsequently influence parent–child relationship quality.

Based on this evidence, Hypothesis 4 (H4) is proposed: Parent–child relationship and exercise behavior jointly exert a chain mediating effect in the association between family physical activity environment and high school students’ mental health.

Rather than presenting disparate theoretical frameworks, this review has systematically built upon Family Functioning Theory as the foundational framework, while strategically incorporating complementary theories to address specific mechanistic pathways. The literature review has primarily articulated the hypothesized directional paths from family physical activity environment to mental health outcomes through various mediating mechanisms. However, contemporary developmental science increasingly recognizes the dynamic and transactional nature of family-adolescent interactions. While attachment-based perspectives emphasize parental influence on child development, substantial evidence also demonstrates that adolescent characteristics—including psychological dispositions, behavioral patterns, and mental health status—can significantly shape parenting practices and family relationship quality (14, 15). Acknowledging this bidirectionality does not contradict the current hypotheses but rather situates them within a broader, dynamic framework. The present study thus focuses on the predominant directional pathway (FPAE → P–CR/EB → MH) supported by existing cross-sectional evidence, while recognizing that longitudinal or experimental designs are required to clarify potential feedback effects.

## 3 Method

### 3.1 Participants and procedure

A stratified sampling method was employed by categorizing cities in Shandong Province based on their type, size, and function. One high school was randomly selected from each of the 16 cities. Within each school, one class from each grade level (Grade 10 to Grade 12) was randomly chosen. The participants were high school students with an average age of 16.33 years (SD = 1.92). Questionnaires were administered collectively during students’ self-study sessions. To ensure informed consent and voluntary participation, students were informed beforehand about the confidentiality measures and the purpose of data collection. Homeroom teachers assisted in the administration process, reminding students not to omit any information. Questionnaires were collected after 45 min. A total of 2,200 questionnaires were distributed, with 151 invalid questionnaires excluded. The final valid sample consisted of 2,049 students, yielding a valid response rate of 93.13%. Among them, 1,106 were male (54.4%) and 943 were female (45.6%).

### 3.2 Measures

#### 3.2.1 Family physical activity environment questionnaire

The Family Physical Activity Environment subscale from the Shanghai Adolescents’ Fitness and Exercise Survey Questionnaire developed by Hu (26) was used to measure the supportive atmosphere of family physical activity. Previous empirical studies have demonstrated that this scale has sound psychometric properties and has been successfully applied in diverse Chinese adolescent samples, indicating its good generalizability and cultural adaptability (27, 28). The subscale consists of four items (e.g., “My parents often exercise with me”). Responses were dichotomous (“yes” = 1, “no” = 0), with higher scores indicating better family physical activity support provided by either or both parents.

Internal consistency of this scale has been well-established, with reported Cronbach’s *α* values typically exceeding 0.85 across different adolescent samples. In the present study, Cronbach’s α coefficients for the overall scale, parental role modeling, and parental support dimensions were 0.93, 0.88, and 0.91, respectively. Construct validity was satisfactory: χ^2^/df = 2.66, RMSEA = 0.04, CFI = 0.99, GFI = 0.98, TLI = 0.99, IFI = 0.99. Convergent and discriminant validity were also acceptable. For the parental role modeling dimension: AVE = 0.52, CR = 0.76, and √AVE = 0.72 (greater than the inter-dimension correlation coefficient of 0.68). For the parental support dimension: AVE = 0.66, CR = 0.80, and √AVE = 0.81 (greater than 0.68).

#### 3.2.2 Mental health diagnostic test

The Mental Health Diagnostic Test (MHT) was adapted from the Anxiety Tendency Diagnostic Test originally translated and revised by Zhou (29), with supplementary items developed with reference to contemporary measurement frameworks including the Mental Health Assessment Inventory (17, 18)and the Multidimensional Anxiety Screening Tool (30). This integrated approach ensures comprehensive coverage of both established and emerging mental health dimensions while maintaining cross-measure comparability. The MHT has undergone continuous refinement and systematic revisions over the past three decades in response to evolving adolescent mental health challenges. Subsequent updates have incorporated emerging psychosocial stressors, such as appearance-related anxiety, academic competition pressure, and social media–induced anxiety, ensuring that the instrument remains aligned with contemporary adolescent psychological characteristics and public health issues. As a result, the MHT has been widely adopted in national mental health surveillance, school-based psychological screenings, and peer-reviewed research across China, with substantial evidence supporting its robust psychometric performance and applicability in diverse adolescent populations. The test consists of 100 items (e.g., “Do you often think about tomorrow’s schoolwork when you try to sleep at night?”) and includes eight content subscales—learning anxiety, interpersonal anxiety, loneliness, self-blame, sensitivity, somatic symptoms, phobic tendencies, and impulsivity—plus one validity subscale (not included in the total score). Responses were scored dichotomously (“yes” = 0, “no” = 1), with higher scores indicating better mental health. Across multiple waves of empirical studies, the MHT has consistently demonstrated solid internal consistency (Cronbach’s *α* typically ranging from 0.85 to 0.95) and strong criterion-related validity, as evidenced by its significant correlations with subjective well-being, school adaptation, and behavioral adjustment among adolescents. In the present study, Cronbach’s *α* reached 0.92. Structural validity indices were also satisfactory: χ^2^/df = 2.67, RMSEA = 0.04, CFI = 0.99, GFI = 0.98, TLI = 0.99, IFI = 0.99. These results further confirm the MHT as a psychometrically sound, age-relevant, and highly practical tool for evaluating adolescent mental health in modern public health contexts. Although the MHT was originally developed in the early 1990s, it has been continuously revised and widely used in large-scale mental health surveys and school-based screenings in China, demonstrating robust psychometric properties and cultural appropriateness for Chinese adolescent populations (29, 31). Its continued application in contemporary research reflects its established reliability and relevance within the Chinese educational context.

#### 3.2.3 Parent–child relationship scale

The Parent–Child Communication Scale developed by Pianta and Virginia (32) and translated into Chinese by Zhang et al. (33) was employed to assess parent–child relationship quality. This scale has been widely used in empirical studies involving Chinese children and adolescents, including research published in peer-reviewed journals on family functioning, parent–child attachment, and adolescent socio-emotional development, demonstrating its maturity and extensive applicability in the Chinese context (12, 13, 34). The Chinese version of the Parent–Child Relationship Scale (33) has been extensively validated and applied in family studies involving Chinese adolescents (12, 13, 34). Its continued use in recent peer-reviewed research attests to its cross-temporal relevance and measurement stability in capturing parent–child dynamics within Chinese familial contexts. The scale consists of 15 items across two dimensions: closeness (7 items) and conflict (8 items). Closeness items were scored positively, whereas conflict items were reverse-coded. Higher total scores indicated better communication between parents and children. Responses were scored dichotomously (“yes” = 1, “no” = 0). Cronbach’s *α* was 0.91. Structural validity indices were satisfactory: χ^2^/df = 2.98, RMSEA = 0.04, CFI = 0.95, GFI = 0.91, TLI = 0.94, IFI = 0.95.

#### 3.2.4 Physical activity rating scale

Students’ exercise behavior was assessed using the Physical Activity Rating Scale (PARS-3) revised by Liang (35). The PARS-3 remains one of the most commonly used physical activity assessments in Chinese adolescent research due to its brevity, ease of administration, and established criterion validity in prior national youth fitness surveys (9, 35). While newer accelerometer-based measures exist, the PARS-3 provides a practical, low-cost tool for large-scale school-based studies where objective monitoring is not feasible. The scale consists of four items (e.g., “What is the intensity of your physical exercise?”), assessing exercise intensity, duration, and frequency. The total exercise score is calculated as intensity × duration × frequency. Intensity and frequency are rated on a 5-point scale (1–5), while duration is rated from 0 to 4. The total score ranges from 0 to 100, with ≤19 representing low exercise level, 20–42 moderate, and ≥43 high. In this study, the Cronbach’s *α* of the scale was 0.80.

### 3.3 Data analysis

Data were analyzed using SPSS 26.0. Pearson correlation analysis, regression analysis, and chain mediation analysis were conducted. The mediation effects were examined using the PROCESS macro (version 4.1) with the Bootstrap method (5,000 resamples). Specifically, Model 6 was selected, with family physical activity environment as the independent variable (X), parent–child relationship as the first mediator (M1), exercise behavior as the second mediator (M2), and mental health as the dependent variable (Y).

## 4 Results

### 4.1 Common method bias test

To minimize potential common method bias, an anonymous coding procedure was adopted during data collection to control for bias at the procedural level. In addition, Harman’s single-factor test was conducted through exploratory factor analysis using SPSS 26.0. The results showed that the first factor accounted for 22.50% of the total variance, which was below the critical threshold of 40%, indicating that common method bias was not a major concern in this study.

### 4.2 Descriptive statistics and correlation analysis

This study focused on the overall scores of the key constructs rather than examining sub-dimensions. Pearson correlation analysis was conducted based on the mean scores of each variable. Table 1 presents the means, standard deviations, and bivariate correlations of the study variables (*N* = 2,049). Overall, students reported a moderate level of family physical activity environment (M = 37.26, SD = 14.46) and parent–child relationship quality (M = 26.11, SD = 11.60). Exercise behavior levels were relatively low to moderate (M = 28.72, SD = 25.31), while mental health scores also fell within the moderate range (M = 55.62, SD = 16.43). As shown in Table 1, all key variables were significantly and positively correlated. Specifically, a more supportive family physical activity environment was associated with better parent–child relationships (*r* = 0.51, *p* < 0.001), higher levels of exercise behavior (*r* = 0.41, *p* < 0.001), and better mental health (*r* = 0.45, *p* < 0.001). Parent–child relationship quality also demonstrated positive correlations with exercise behavior (*r* = 0.42, *p* < 0.001) and mental health (*r* = 0.47, *p* < 0.001). In addition, exercise behavior was moderately and positively correlated with mental health (*r* = 0.44, *p* < 0.001).

**Table 1:** Means, standard deviations, and correlation coefficients of study variables (*n* = 2049).

Variable	M	SD	1	2	3	4
1. Family physical activity environment	37.26	14.46	1			
2. Parent–child relationship	26.11	11.60	0.51^***^	1		
3. Exercise behavior	28.72	25.31	0.41^***^	0.42^***^	1	
4. Mental health	33.75	27.83	0.45^***^	0.47^***^	0.44^***^	1

*p < 0.05, **p < 0.01, ***p < 0.001.

### 4.3 Direct and indirect effects of family physical activity environment on mental health

Given the significant correlations among family physical activity environment, parent–child relationship, exercise behavior, and mental health, the data met the statistical requirements for testing both direct and indirect effects. The PROCESS macro in SPSS was employed, controlling for demographic variables (gender and grade), with 5,000 bootstrap samples set at a 95% confidence interval to examine the chain mediation effects of parent–child relationship and exercise behavior between family physical activity environment and mental health.

First, multiple regression analysis demonstrated that family physical activity environment significantly predicted mental health directly (*β* = 0.46, *p* < 0.001). Even after including family physical activity environment, parent–child relationship, and exercise behavior in the regression model simultaneously, the predictive effect of family physical activity environment on mental health remained significant (*β* = 0.23, *p* < 0.001). Moreover, family physical activity environment significantly predicted parent–child relationship (*β* = 0.50, *p* < 0.001) and exercise behavior (*β* = 0.27, *p* < 0.001). Parent–child relationship significantly predicted exercise behavior (*β* = 0.29, *p* < 0.001) and mental health (*β* = 0.26, *p* < 0.001). Exercise behavior also significantly predicted mental health (*β* = 0.24, *p* < 0.001; see Table 2).

**Table 2:** Regression analysis of relationships among model variables.

Regression equation		Overall fit index			Regression coefficient significance			*p*
Outcome variable	Predictor variable	R	R^2^	F	β	SE	t	
Mental health	Gender				0.02	1.54	0.64	0.524
	Grade	0.45	0.21	90.68	−0.01	1.54	−0.48	0.631
	FPAE				0.46	0.05	16.46^***^	<0.001
Parent–child relationship	Gender				−0.02	0.63	−0.77	0.442
	Grade	0.50	0.25	115.18	−0.04	0.62	−1.30	0.194
	FPAE				0.50	0.02	18.58^***^	<0.001
Exercise behavior	Gender				−0.02	0.62	−0.65	0.518
	Grade	0.42	0.18	443.72	−0.03	0.62	−1.12	0.262
	P–C R				0.29	0.07	9.16^***^	<0.001
Exercise behavior	Gender				−0.00	1. 44	−0.08	0.934
	Grade	0.48	0.23	78.01	−0.01	1.44	−0.41	0.682
	FPAE				0.27	0.06	8.53^***^	<0.001
	P–C R				0.29	0.07	9.16^***^	<0.001
Mental health	Gender				0.03	1. 42	0.98	0.327
	Grade				0.00	1.42	0. 02	0.358
	FPAE	0.57	0.33	101.31	0.23	0.06	7.6 l^***^	<0.001
	P–C R				0.26	0.07	8.36^***^	<0.001
	E B				0.24	0.03	8.1 L^***^	<0.001

*p < 0.05, **p < 0.01, ***p < 0.001. Parent–Child Relationship abbreviated as P–C R; Exercise Behavior abbreviated as E B; Family Physical Activity Environment abbreviated as FPAE.

**Figure 1:**
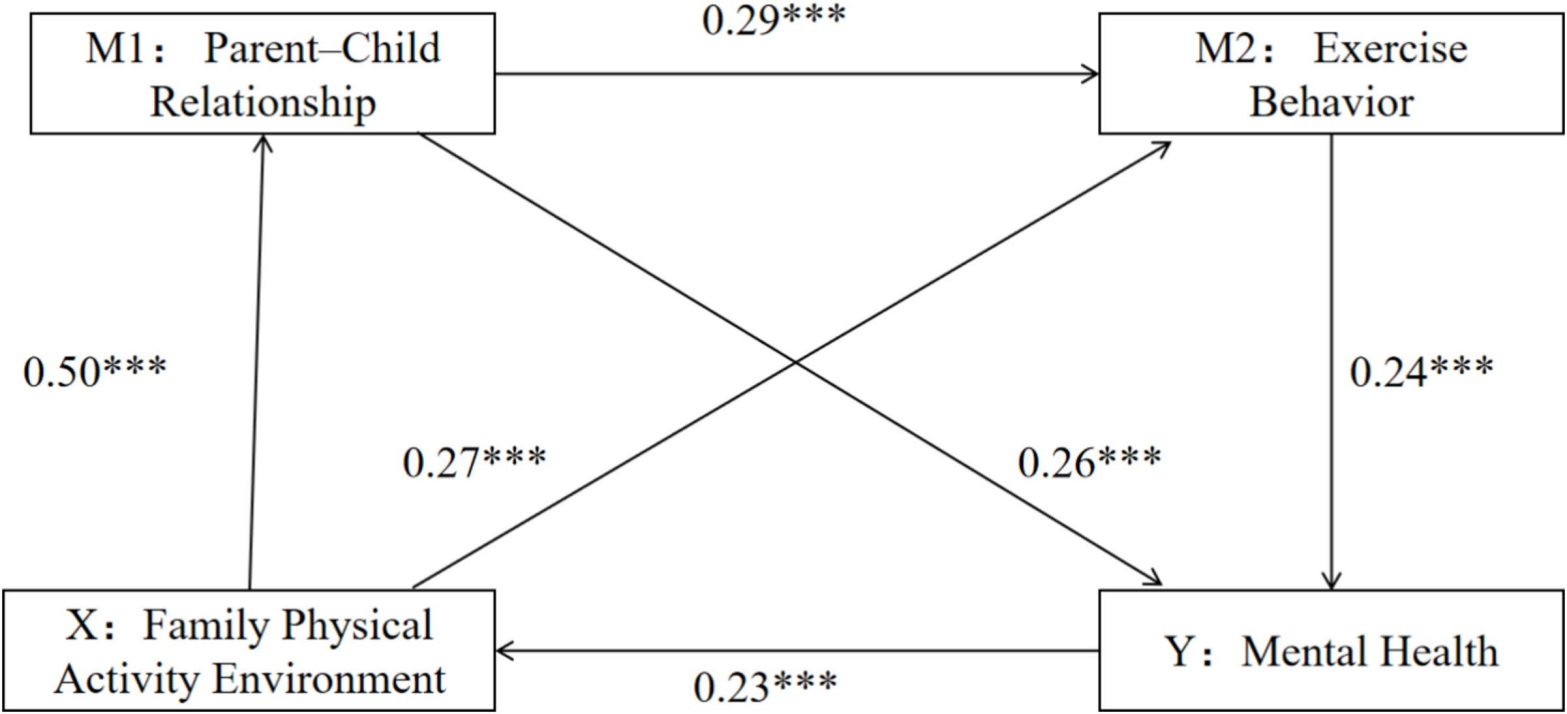
The pathway of the impact of family physical activity environment on mental health.

Second, mediation analysis was conducted. As shown in Table 3, both parent–child relationship and exercise behavior mediated the association between family physical activity environment and mental health, with a total indirect effect of 0.43. The 95% confidence interval [0.34, 0.52] excluded zero, indicating a significant mediation effect, which accounted for 49.20% of the total effect of family physical activity environment on mental health (0.88). The mediation effect consisted of three indirect pathways:family physical activity environment → Parent–child relationship → Mental health: indirect effect = 0.25; the 95% confidence interval excluded zero, confirming significance, explaining 27.97% of the total effect (0.88).family physical activity environment → Exercise behavior → Mental health: indirect effect = 0.12; the 95% confidence interval excluded zero, confirming significance, explaining 13.81% of the total effect (0.88).family physical activity environment → Parent–child relationship → Exercise behavior → Mental health: indirect effect = 0.07; the 95% confidence interval excluded zero, confirming significance, explaining 7.42% of the total effect (0.88). The final mediation model illustrating the pathways from family physical activity environment to mental health is presented in Figure 1.

**Table 3:** Bootstrap results of mediation effect analysis.

Effect	Effect value	Boot SE	95% CI		Effect ratio
			Lower limit	Upper limit	
Indirect effect 1	0.25	0.04	0.17	0.32	27.97%
Indirect effect 2	0.12	0.03	0.07	0.18	13.81%
Indirect effect 3	0.07	0.01	0.04	0.10	7.42%
Total indirect effect	0.43	0.05	0.34	0.52	49.20%
Direct effect	0.45	0.06	0.33	0.56	50.80%
Total effect	0.88	0.05	0.77	0.98	100.000%

## 5 Discussion

This study revealed both the direct and indirect pathways through which the family physical activity environment influences the mental health of high school students, representing a positive exploration into the prevention and promotion of adolescent mental health. Theoretically, it enriches the literature on the influencing factors and mechanisms of mental health, while further advancing research on family physical activity environment. Practically, it highlights the significance of family physical activity environment, offering new perspectives for enhancing parent–child relationships, cultivating exercise habits, and reducing mental health problems among students. Furthermore, this study provides actionable implications for key stakeholders—including parents, schools, and policymakers—on how to cultivate a supportive family physical activity environment and translate its benefits into improved mental health outcomes.

### 5.1 Direct effects of the family physical activity environment on mental health

The findings show that the family physical activity environment has a significant positive effect on students’ mental health, with an effect size of 0.45, thereby confirming Hypothesis H1. In this study, the direct effect of the family physical activity environment on mental health was greater than its indirect effect (0.43), underscoring its central role in shaping adolescent mental health. The family physical activity environment serves as the first medium through which individuals engage in physical activity and functions as an effective means of promoting physical and psychological well-being. The findings show that the family physical activity environment has a significant positive effect on students’ mental health, with an effect size of 0.45, thereby confirming Hypothesis H1. It is noteworthy that nearly half of the total effect (49.20%) was mediated through parent–child relationships and exercise behavior, indicating that the indirect pathways are almost as influential as the direct effect. This pattern suggests that while the family physical activity environment directly shapes adolescent mental health, its impact is substantially amplified through relational and behavioral mechanisms.

The direct influence of the family physical activity environment can be interpreted from two dimensions: modeling and family support. From the perspective of social learning theory, parental modeling explains the influence of observational learning on children’s mental health. Parents who maintain regular exercise habits not only signal a healthy psychological state but also provide “vicarious reinforcement” for their children, indirectly enhancing their mental well-being. From the perspective of social support theory, parental support exerts a direct influence on adolescents’ mental health. The family physical activity environment fulfills both objective needs (material and social resources) and subjective needs (cognition, emotion, self-esteem). Through understanding, care, and affection, parents provide adolescents with a sense of security and acceptance, which contributes to the development of a positive self-identity and, in turn, fosters improvements in mental health.

### 5.2 The mediating role of parent–child relationships

This study further demonstrates that the effect of the family physical activity environment on mental health can also be mediated through parent–child relationships, thereby confirming Hypothesis H2. This finding is consistent with previous research on the predictive role of parent–child relationships in mental health (8). According to family functioning theory, the fundamental function of the family is to provide appropriate conditions for members’ physical and psychological development. As a key manifestation of family functioning, the parent–child relationship serves not only as an emotional bond but also as a determinant of children’s developmental outcomes, including their mental health.

Attachment theory posits that adolescents’ relationships with parental figures extend the influence of family functioning (15). During adolescence, students gradually transition from dependence and compliance to autonomy and individuation. While striving for independence, they still require parental understanding and support, underscoring the importance of effective communication. This study shows that a supportive family physical activity environment promotes positive parent–child relationships. Positive communication in sports-related family education—such as encouragement, support, and praise—can be more readily accepted by adolescents, stabilizing family relationships and ultimately promoting mental health.

### 5.3 The mediating role of exercise behavior

The findings also indicate that the family physical activity environment affects mental health indirectly by promoting exercise behavior, supporting Hypothesis H3. This result aligns with previous studies highlighting the predictive role of exercise behavior in mental health (36). Participation in physical activity strengthens the neural circuits involved in self-regulation, thereby enhancing cognitive control, self-concept, and problem-solving abilities (37).

Given that high school students are surrounded by electronic devices and online media from an early age, their sedentary behaviors have increased while physical activity has declined, leading to reduced physical and mental health. Intergenerational transmission theory suggests that parents’ beliefs, attitudes, and behaviors influence children’s motivation to engage in physical activity (38). Supportive parental actions—such as encouragement, modeling, and financial investment—stimulate children’s enthusiasm for exercise. In contrast, negative parental behaviors, such as excessive screen time, provide poor role models and hinder the development of healthy habits. These findings underscore the critical role of the family physical activity environment in fostering adolescent mental health.

### 5.4 The chain mediating role of parent–child relationships and exercise behavior

Beyond their independent mediating effects, parent–child relationships and exercise behavior jointly serve as a chain mediator in the association between the family physical activity environment and adolescent mental health, supporting Hypothesis H4. This finding indicates that a supportive family physical activity environment not only shapes adolescents’ behavioral patterns but also exerts influence through family relational and psychological motivational pathways.

First, family functioning theory provides a foundation for explaining why the family physical activity environment promotes mental health through parent–child relationships. A positive physical activity environment reflects effective family functioning, characterized by emotional responsiveness, communication, and cohesion. When parents participate in physical activity with their children, they model supportive behaviors, enhance family interaction quality, and foster secure attachment and emotional connectedness, which in turn elevate adolescents’ psychological well-being. Second, based on self-determination theory, physical activity engagement driven by parental support satisfies adolescents’ basic psychological needs for autonomy, competence, and relatedness. A healthy family physical activity environment promotes adolescents’ internal motivation to exercise, turning physical activity from an externally enforced task into a self-endorsed behavior (39). This internalization process not only strengthens exercise habits but also contributes to improved emotional regulation and mental health. The integration of family functioning theory and self-determination theory therefore explains the “why” behind the chain pathway: the family context enhances relationship quality (parent–child relationship), which then stimulates intrinsic motivation for physical activity and increases exercise participation, ultimately benefiting mental health.

The chain mediation model (FPAE → Parent–Child Relationship → Exercise Behavior → Mental Health) reveals a sequential process where the family physical activity environment first enhances relationship quality (*β* = 0.50), which subsequently promotes exercise behavior (*β* = 0.29), ultimately contributing to mental health (*β* = 0.24). This “family–individual” and “physical health–mental health” positive feedback loop is empirically supported by the significant path coefficients, demonstrating how relational improvements precede and enable behavioral changes (25). Within this system, the family physical activity environment operates as a subsystem where parental involvement in physical activity nurtures children’s exercise routines, strengthens parent–child bonds, and enhances emotional support, ultimately contributing to adolescents’ sense of safety and mental health. Compared with previous research, which mainly emphasized the direct impact of the parent–child relationship or exercise behavior on mental health, the present study reveals a more comprehensive and sequential mechanism. Prior studies typically examined these mediators separately, while our findings confirm that the parent–child relationship precedes and enhances exercise behavior, forming a chain effect. This extends earlier work by demonstrating that a positive family physical activity environment not only benefits adolescents’ physical and psychological development independently but also amplifies mental health gains through this sequential pathway. Overall, testing this chain mediation model deepens the theoretical understanding of how the family physical activity environment shapes adolescent mental health and highlights the importance of enhancing both family functioning and intrinsic exercise motivation in intervention practices.

### 5.5 Practical implications for stakeholders

Given that this study was conducted within Shandong Province, characterized by specific socioeconomic and educational contexts, the following implications should be considered as potentially context-specific. Future interventions may require adaptation based on local educational priorities and resource availability.

First, parents should actively participate in physical activities with their children and provide autonomy-supportive encouragement rather than coercive control. Establishing regular family exercise routines (e.g., weekend hiking, family sports games) can strengthen emotional bonds and facilitate intrinsic motivation for exercise.

Second, schools should integrate family-based physical activity programs into health education curricula and parent–school collaboration initiatives. For example, organizing parent–child sports events, providing informational workshops on the importance of family physical activity, and offering guidance to parents on how to create supportive home environments could enhance the joint involvement of families and schools. Third, educational and health authorities should develop policies that encourage family involvement in physical activity, such as community-based family sports programs, public health campaigns promoting family exercise, and financial or resource support for low-income families to access sports facilities. Establishing interdepartmental collaborations between education, health, and community sectors may further scale up the impact of family physical activity promotion on adolescent mental health.

### 5.6 Limitations and future directions

Although this study offers valuable insights, several limitations should be noted to guide interpretation and future research.

#### 5.6.1 Limitations

First, the study focused on a limited set of variables within the family physical activity context. While the model captured key mechanisms, it did not examine other potentially relevant family or individual factors (e.g., parenting style, peer influence), which may also contribute to adolescents’ mental health and behavioral outcomes.

Second, the generalizability of findings may be constrained by the regional sampling frame. As the study was conducted in Shandong Province, the “critical factor” role of family physical activity environment identified here might reflect context-specific demographic, socioeconomic, and educational realities that differ from other regions in China. Third, this study primarily focused on the holistic construct of the family physical activity environment and did not delve into the specific mechanisms of its sub-dimensions (e.g., role modeling, psychological support). Future research examining the differential pathways of these dimensions could provide a more refined understanding of how family physical activity environments influence adolescent mental health.

#### 5.6.2 Future directions

First, future studies could extend the model by integrating additional family or psychosocial variables (e.g., resilience, parent–child interaction quality) to develop more comprehensive explanatory frameworks. Second, adopting multi-informant and multi-method assessments—such as parent or teacher reports, behavioral observations, or wearable-based activity tracking—would reduce subjective bias and enhance ecological validity. Third, expanding sampling to multiple regions and demographic groups would improve generalizability. Comparative studies across urban–rural settings or diverse family structures could help identify variations and inform more targeted public health strategies.

## 6 Conclusion

This study yields two principal findings regarding high school students. First, family physical activity environment, parent–child relationship, exercise behavior, and mental health demonstrate significant interrelationships. Second, the family physical activity environment serves as a critical determinant of adolescent mental health, exerting both direct effects and indirect effects through the mediating roles of parent–child relationships and exercise behavior. Most importantly, these findings carry compelling practical implications: interventions aimed at promoting adolescent mental health should extend beyond merely encouraging physical activity per se. Instead, they should specifically target the enhancement of parent–child communication and relational quality through structured joint physical activities. Given the demonstrated significance of the parent–child relationship pathway, fostering shared physical experiences within families emerges as a crucial strategy for amplifying the mental health benefits derived from the family physical activity environment.

## Data Availability

The original contributions presented in the study are included in the article/supplementary material, further inquiries can be directed to the corresponding author.
